# NELL2 as a potential marker of outcome in the cerebrospinal fluid of patients with tuberculous meningitis: preliminary results from a single-center observational study

**DOI:** 10.1186/s40001-022-00921-7

**Published:** 2022-12-09

**Authors:** Jianhua Chen, Jie Wu, Yong Luo, Nanqu Huang

**Affiliations:** 1 Department of Neurology, Third Affiliated Hospital of Zunyi Medical University, (The First People’s Hospital of Zunyi), Zunyi, 563000 China; 2 Scientific Research Center, Third Affiliated Hospital of Zunyi Medical University, (The First People’s Hospital of Zunyi), Zunyi, 563000 China; 3 National Drug Clinical Trial Institution, Third Affiliated Hospital of Zunyi Medical University, (The First People’s Hospital of Zunyi), Zunyi, 563000 China

**Keywords:** Tuberculous meningitis, Nel-like 2, Noncentral nervous system infection, Cerebrospinal fluid

## Abstract

**Objective:**

To detect the changes in Nel-like 2 (NELL2) in cerebrospinal fluid (CSF) in the outcome of tuberculous meningitis (TBM) patients and to initially evaluate its potential as a marker.

**Methods:**

We collected the clinical data of patients with suspected TBM in the First People’s Hospital of Zunyi from November 2017 to January 2021 and retained their CSF. According to the selection and exclusion criteria, the TBM group (11 cases) and the control group (18 cases) were obtained. Western blotting (WB) was used to detect the level of NELL2 in the CSF of the two groups, especially the change in NELL2 before and after treatment in TBM patients.

**Results:**

The level of NELL2 in the TBM group was lower than that in the control group (*P* < 0.05), and the level of NELL2 showed an increasing trend after anti-tuberculosis treatment in the TBM group.

**Conclusions:**

NELL2 in the CSF of TBM patients decreased significantly. Anti-tuberculosis treatment can improve the level of NELL2, which may become one of the potential markers of outcome in the cerebrospinal fluid of patients with tuberculous meningitis.

## Introduction

Tuberculosis meningitis (TBM) is a type of tuberculosis (TB) that involves the meninges after infection by Mycobacterium tuberculosis (MTB). This disease is a common extrapulmonary tuberculosis and the most serious of various tuberculosis diseases [1]. In recent years, the prevalence of TB has increased due to HIV infection, excessive use of anti-tuberculosis drugs, and the emergence of drug-resistant tuberculosis bacteria [2, 3]. However, the early clinical manifestations of TBM, cerebrospinal fluid (CSF) routine and biochemical results lack specificity and are not typical in the early stages of TBM. When the patient’s neurological symptoms are obvious, the patient may have permanent sequelae [2]. The prognosis of TBM patients is related to their age and disease severity. If the patient’s symptoms disappear and the CSF examination is normal, it indicates a good prognosis, but a small number of patients will still die after the correct treatment. Therefore, the outcome of TBM cannot be evaluated solely on the basis of the clinical manifestations of patients. Exploring the outcome markers of TBM, which can effectively increase the cure rate, reduce the mortality rate, and reduce the rate of disability, is also necessary.

Nel-like 2 (NELL2) is a neuron-specific secretory protein [4]. Early studies have found that NELL2 can promote the differentiation and survival of neural cells during embryonic development through mitogen-activated protein kinase and can also promote the differentiation of neural progenitor cells into mature neurons [5]. Relevant studies have shown that the level of NELL2 in the normal control group is significantly higher than that in the TBM group. NELL2 may be a potential biomarker for the early diagnosis of TBM [6]. However, whether the dynamic changes in NELL2 can be used as a marker of TBM outcome remains to be further studied. However, the study did not show a difference in NELL2 in other viral, fungal, and bacterial meningitis, nor did it dynamically observe changes in NELL2 levels after anti-tuberculosis treatment in the TBM disease group. Therefore, in this study, the differences in NELL2 in CSF between the TBM disease group and the control group and the changes in NELL2 in the TBM group after anti-tuberculosis treatment were explored by Western blotting.

## Materials and methods

### Methods and study protocol

**Fig. 1:**
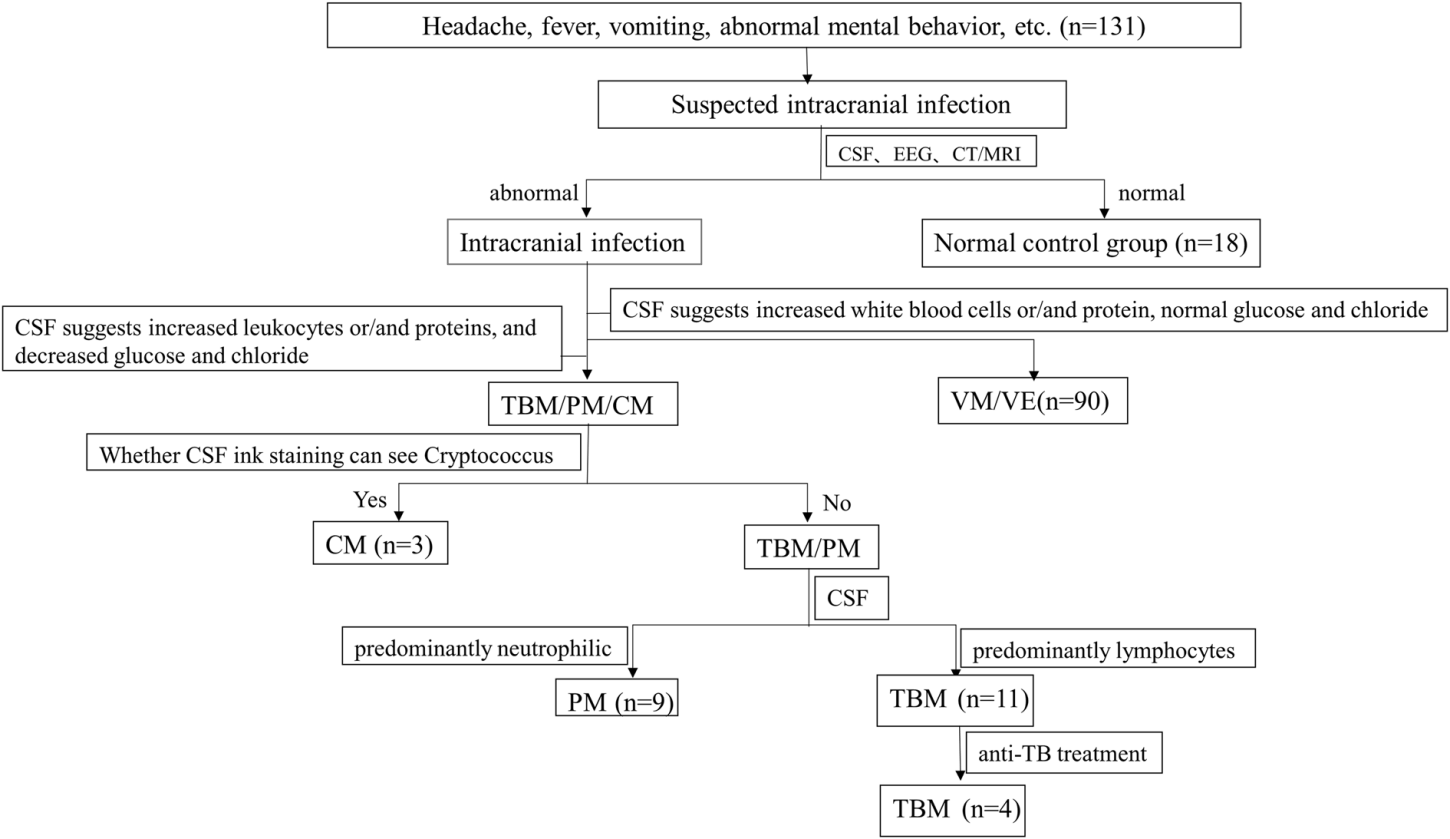
Participant flowchart. *TBM* tuberculous meningitis, *PM* purulent meningitis, *CM* cryptococcal meningitis, *VM/VE* viral meningitis/encephalitis

This study protocol was approved by the Ethics Committee of the First People's Hospital of Zunyi. A total of 131 patients from the Department of Neurology and Infectious Diseases, The First People’s Hospital of Zunyi, were identified, screened, and enrolled in the study between November 2017 and January 2021, and all patients provided informed consent (Fig. 1).

### Inclusion/exclusion criteria

Patients in this study were recruited from a population of suspected TBM patients and were invited to participate in the study if they fulfilled all of the following criteria. Inclusion criteria for the TBM group [7–9]: (1) symptoms of tuberculosis poisoning such as low-grade fever, night sweats, weight loss, general malaise, or a history of exposure to tuberculosis; (2) fever, vomiting, and positive meningeal irritation signs; (3) CSF pressure increased, the number of white blood cells (mainly lymphocytes) in CSF was 50–500 × 10^6^/L, protein increased, glucose and chloride decreased; (4) *Mycobacterium tuberculosis* DNA test positive in CSF; (5) head CT or MRI examination showing basal cistern exudation or hydrocephalus or cerebral infarction; (6) anti-tuberculosis treatment is effective; (7) provided informed consent. Inclusion criteria for the control group: (1) no history of contact with tuberculosis or history of the previous infection with tuberculosis or herpes; (2) patients with clinical symptoms such as dizziness, headache, and fever, and suspected central nervous system infection, requiring lumbar puncture to obtain CSF for testing; (3) CSF results were normal and EEG showed no slow waves or spikes; (4) neurological examination was unremarkable; (5) no cerebrovascular accident, brain tumor and central nervous system infectious diseases; (6) effective symptomatic treatment (not anti-tuberculosis); (7) provided informed consent.

Exclusion criteria for the TBM group was as follows: (1) cryptococcal meningitis, purulent meningitis, viral meningitis; (2) intracranial tumor; (3) those who had taken anti-tuberculosis drugs before admission. Exclusion criteria for the control group was as follows: (1) neurological examination showed meningeal irritation, but head CT/MRI showed cerebrovascular accident and brain tumor; (2) CSF results were abnormal; (3) CT of chest and abdomen suggests tuberculosis or pulmonary infection.

### CSF collection

Six to eight milliliters of CSF were collected from the lumbar 4–5 intervertebral space by lumbar puncture and centrifuged at 4 ℃ for 15 min at 1200 rpm/min, and the supernatant was aliquoted and stored in a – 80 ℃ refrigerator. For patients diagnosed with TBM, after anti-tuberculosis treatment, the CSF of the same patient was collected again and stored in a – 80 ℃ refrigerator.

### Western blot assay

The CSF was lysed and centrifuged, and the protein in the supernatant was quantified using a BCA kit. An equal amount of total protein was added to a 10% Bis–Tris Nu-PAGE gel. The voltage was set to 90 V, and electrophoresis was run for 15 min. When the sample was in the same position, the voltage was set to 100 V, and electrophoresis was run for 90 min. The membrane was transferred at 300 mA for 35 min. Then, the membrane was blocked with 5% skim milk, incubated with NELL2 (1:3000) at 4 ℃ overnight, and washed 3 times with TBST. Finally, High-Sig ECL Western Blotting Substrate (Shanghai Tanon Technology Co., Ltd.) was used to visualize the membrane.

### Statistical analysis

All statistical analyses were performed with SPSS 18.0 (IBM, USA). The measurement data that have a normal distribution are represented as the mean ± SD. Those data that did not have a normal distribution are expressed as *P50* (*P25* ~ *P75*). The *t*-test was used to compare the data with a normal distribution and homogeneity of variance, and the rank sum test was used for the nonnormal data. The differences before and after anti-tuberculosis treatment in the TBM group were analyzed by repeated measures analysis of variance.

## Results

### General clinical information

According to the inclusion and exclusion criteria of each group, 18 patients in the control group and 11 patients with TBM were finally enrolled. The control group was as follows: 6 males and 12 females, with an average age of 50.17 ± 13.75 years. The TBM group was as follows: 5 males and 6 females, average age (46.73 ± 16.19) years. The clinical manifestations in the 11 TBM group were mainly headache in 8 cases, tuberculosis poisoning symptoms (such as fatigue, anorexia, weight loss) in 4 cases, fever and vomiting in 6 cases, and disturbance of consciousness in 2 cases. There were 7 cases with other tuberculosis (6 cases with pulmonary tuberculosis, 1 case with thoracic and lumbar tuberculosis). One case of TB antibody was weakly positive, and the others were negative. Two cases were positive for the *Mycobacterium tuberculosis* DNA test, and 3 cases were weakly positive. Cranial CT or MRI showed hydrocephalus or cerebral infarction in 3 cases. EEG was abnormal in 5 cases. The relevant literature [10] provides the classification of CSF results, as shown in the following table (Table 1).

**Table 1:** Main clinical manifestations of the TBM group and basic information of CSF

Variable	Cases (*n*)	Variable	Cases (*n*)
Clinical manifestations		Protein (0.150–0.45 g/L)	
Headache	8	0.15–0.45	0
Weakness	4	0.45–1.00	0
Fever, vomiting	6	1.00–2.00	2
Unconsciousness	2	> 2.00	9
Other tuberculosis	7	Glucose (2.5–4.4 mmol/L)	
CSF		0.00–1.00	2
The pressure of CSF (80–180 mmH_2_O)		1.01–2.49	8
80–180	1	2.50–4.40	1
180–300	4	Chloride (120–130 mmol/L)	
> 300	6	< 102	3
Number of white blood cells (0–5 × 10^6^/L)		102–116	4
51–200	4	116.1–119.9	2
200–500	5	120.0–130.0	2
≥ 500	2		

### Comparison of NELL2 in CSF in the control group and TBM group

**Fig. 2:**
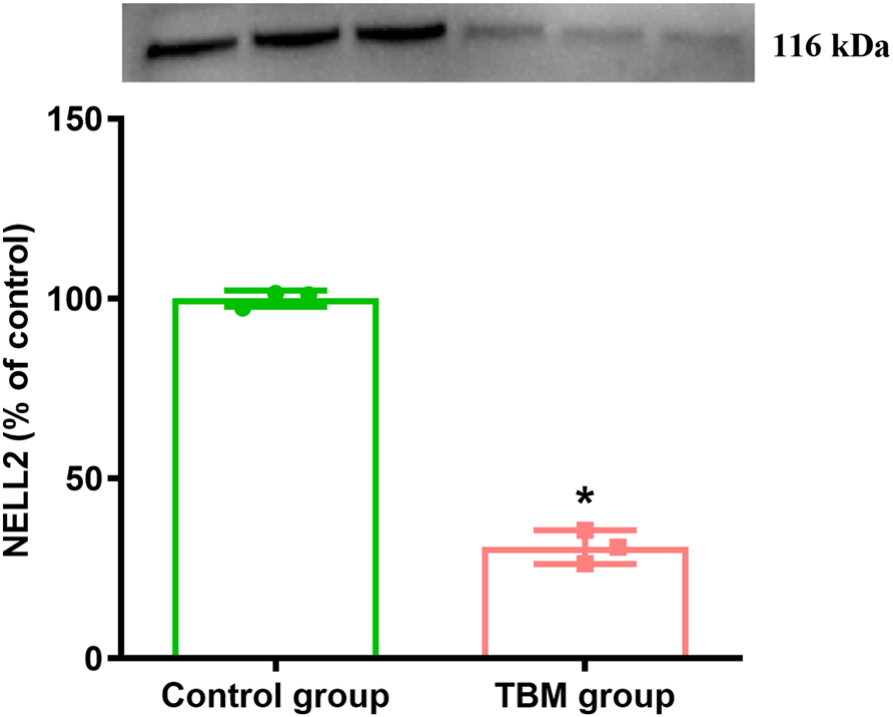
Comparison of NELL2 in CSF between controls and TBM patients. Mean ± SD (^*^P < 0.05 vs. the control group)

In this study, to further observe the differences in NELL2 between the control group and the TBM group, the WB method was used to detect the level of NELL2. The results showed that the level of NELL2 in the TBM group was significantly lower than that in the control group, and the difference was statistically significant (*t* = − 26.23, *P* < 0.01) (Fig. 2).

### Changes in NELL2 levels in CSF after anti-tuberculosis treatment in 4 cases of TBM

**Fig. 3:**
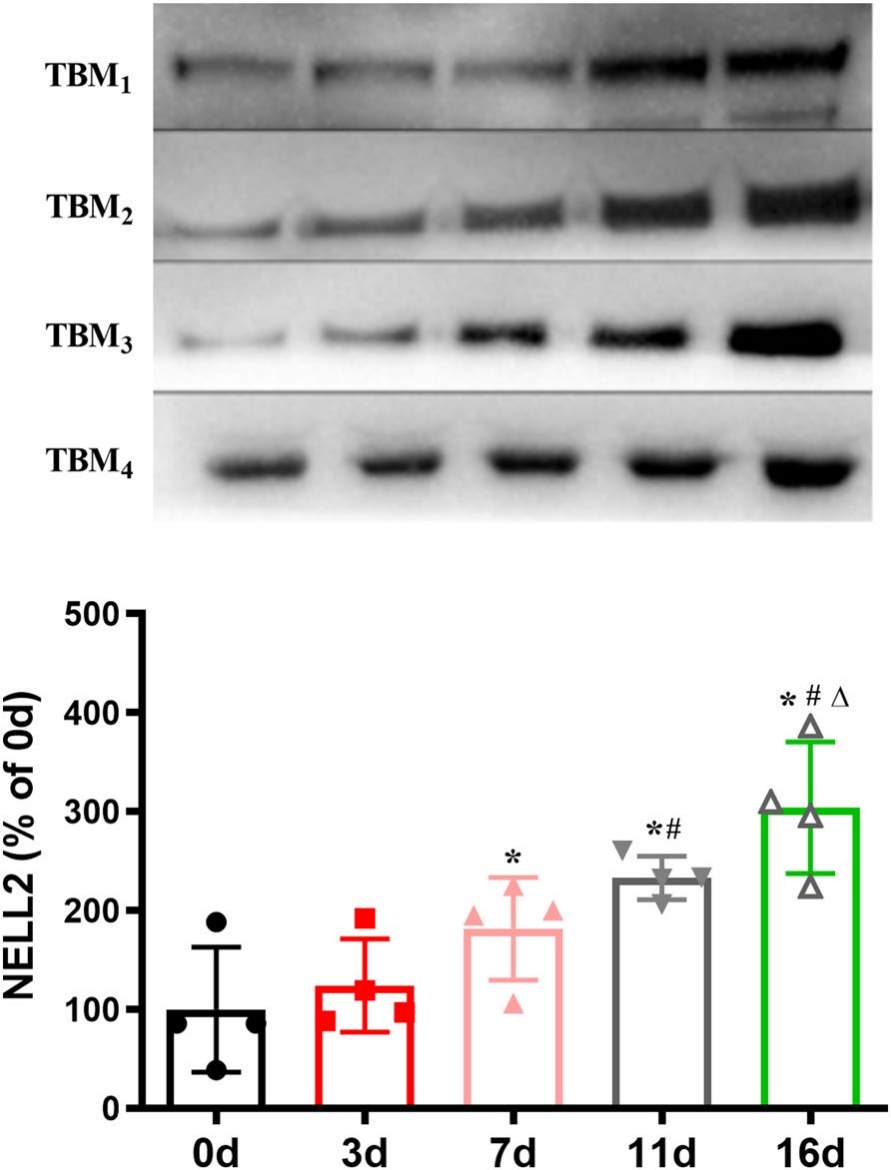
Comparison of NELL2 before and after anti-tuberculosis treatment in TBM patients, mean ± SD. 1, 2, 3, 4 of the TBM represents the number of four different patients (^*^P < 0.05 vs. 0 d, ^#^*P* < 0.05 vs. 3 d, ^△^*P* < 0.05 vs. 7 d)

Among the 11 cases clinically diagnosed with TBM, 5 cases were positive for the *Mycobacterium tuberculosis* nucleic acid DNA test, and 4 cases were followed up 5 times. These four patients were treated with isoniazid, rifampicin, pyrazinamide, and ethambutol for anti-tuberculosis treatment. The clinical symptoms and signs of the patients improved after treatment. To further observe the changes in CSF, CSF was obtained by lumbar puncture at 3 days, 7 days, 11 days, and 16 days after treatment, and the changes at different time points were detected. The results are presented in Table 2 and Fig. 3.

**Table 2:** Comparison of the TBM group before and after anti-tuberculosis treatment

	Leukocyte (× 10^6^/L)	Chloride (mmol/L)	Glucose (mmol/L)	LDH (U/L)	Protein (g/L)
Before therapy	450.00 ± 94.17	110.28 ± 10.79	1.58 ± 0.85	80.90 ± 50.31	2.13 ± 1.30
Treatment for 3 days	314.00 ± 296.60	112.88 ± 10.56	1.73 ± 1.20	83.05 ± 62.05	1.78 ± 1.08
Treatment for 7 days	205.50 ± 190.09	118.30 ± 8.39	1.60 ± 0.80	67.40 ± 69.18	1.27 ± 0.95
Treatment for 11 days	148.75 ± 161.26	118.45 ± 4.94	1.63 ± 0.88	38.58 ± 28.66	0.92 ± 0.54
Treatment for 16 days	122.00 ± 135.23	122.00 ± 3.90	1.75 ± 0.99	26.70 ± 16.87	0.81 ± 0.44
*F* value	5.115	6.830	0.234	4.637	6.922
*P* value	0.084	0.047	0.764	0.098	0.063

### Changes in NELL2 before and after anti-tuberculosis treatment

The levels of NELL2 were low before treatment and tended to increase after anti-tuberculosis treatment (Fig. 3). NELL2 increased at 7 days, 11 days and 16 days after treatment compared with before treatment (*P* < 0.05). NELL2 increased at 11 days and 16 days of treatment compared with 3 days after treatment (*P* < 0.05). NELL2 increased at 16 days of treatment compared with 7 days after treatment (*P* < 0.05).

## Discussion

Auxiliary examinations for TBM diagnosis mainly rely on CSF and imaging examinations. CSF is a substance that the brain constantly produces and exchanges. When infected with TBM, glial cells interact with some components of Bacillus toxins to release inflammatory factors, thereby disrupting the blood–brain barrier and altering the metabolic environment, leading to changes in the composition and properties of CSF. Therefore, CSF can reflect CNS infectious diseases. Detecting changes in CSF indicators is an effective way to assess the outcome of TBM.

NELL2 is a neuron-specific secreted protein that is widely expressed in the brain and is most abundant in neurons of the hippocampus and cerebral cortex [11]. NELL2 plays an important role in neuronal proliferation and differentiation [12]. The content of NELL2 is relatively stable in the brain and changes when neurons are damaged or differentiated. This study found that the level of NELL2 in the TBM group was significantly lower than that in the control group. Interestingly, with anti-tuberculosis drug treatment, not only did the symptoms and signs of the patients improve, but the level of NELL2 also showed a recovery trend. In addition to biochemical indicators, imaging studies are also considerably valuable in diagnosing TBM. Active pulmonary tuberculosis or old tuberculosis can be seen on chest X-ray or chest CT in patients with TBM [13], and symptoms such as hydrocephalus, tuberculous fibrous exudation, and miliary tuberculosis nodules can be found on head CT [14]. In addition, MRI can indicate basal cistern, multifocal enhancement of brain parenchyma, hydrocephalus, and intracranial tuberculosis combined with intraspinal tuberculosis [15]. Based on our research results, we believe that combined with imaging examinations, detecting the dynamic changes of NELL2 will be more accurate for the prognosis of TBM.

## Limitations

However, because TBM has been treated for a long time, in the early stage and other intracranial infectious diseases, it is not effective to observe the changes of the condition only according to the changes of clinical symptoms. Therefore, it is necessary to dynamically recheck lumbar puncture to understand the situation of CSF. However, this is an invasive procedure, so some patients refused lumbar puncture in the late stage, we were unable to collect CSF. In addition, some patients' hospitalization time is too short for financial distress, or they are transferred to other hospitals. The number of cases collected in this study is too small, the tracking time is relatively short. The major limitation in the sample size. Because this is a preliminary clinical study, we are using data meeting the assumption is using the *t*-test. But, nevertheless the statistical power is usually very low with these sample sizes. We can further expand the sample size and extend the follow-up time, hoping to bring certain guiding significance for clinical diagnosis and treatment.

## Conclusions

In conclusion, the dynamic changes in NELL2 can be used as a marker of TBM outcome. However, this study also has certain limitations. For example, the patient’s symptoms improved, and the patient was discharged from the hospital, resulting in a short follow-up time. Therefore, the patient’s symptoms and changes in NELL2 cannot be observed for a longer period of time. In addition, due to the need to collect CSF multiple times, fewer patients agreed to participate in this study, resulting in a small number of cases in this study, which needs to be further expanded for verification.

## Data Availability

The datasets generated or analyzed during this study are available from the corresponding author on reasonable request.
